# Supplementary material for: Modeling unveils sex differences of signaling networks in mouse embryonic stem cells
Source: Mol Syst Biol. 2023 Sep 21;19(11):e11510. doi: 10.15252/msb.202211510 (PMC10632733; doi:10.15252/msb.202211510)
Supplement: Supplementary file 1 — Expanded View Figures PDF [file MSB-19-e11510-s003.pdf]

Expanded View Figures

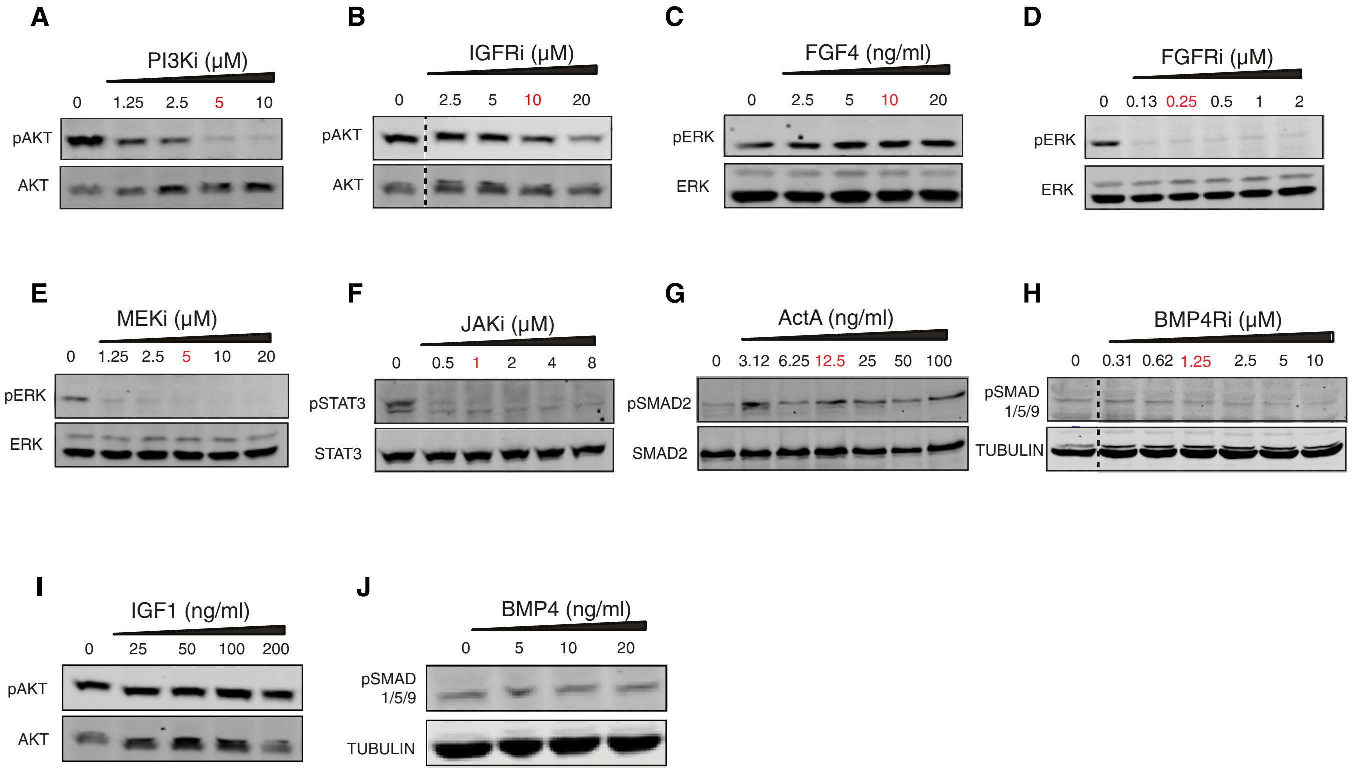

Figure EV1. Testing inhibitor and ligand concentrations to be used for systematic perturbation experiments.

A–J For each perturbation, 1.8 XX mESCs were treated with different concentrations of the treatments for 30 min to select the non-saturating optimal dose. The effect of PI3K inhibitor (LY294002) on pAKT (Ser 473) (A), of IGFR inhibitor (OSI-906/Linsitinib) on pAKT (Ser 473) (B), of FGF4 on pERK (Thr202/Tyr204) (C), of FGFR inhibitor (CH5183284/Debio-1347) on pERK (Thr202/Tyr204) (D), of MEK inhibitor (U0126) on pERK (Thr202/Tyr204) (E), of JAK inhibitor (JAK inhibitor I) on pSTAT3 (Tyr-705) (F), of Activin on pSMAD2 (Ser 465/467) (G), of BMP4 receptor inhibitor (LDN-193189) on pSMAD1/5 (Ser463/465) (H), of IGF1 on pAKT (Ser 473) (I) and of BMP4 on pSMAD1/5 (Ser463/465) (J) was assayed. The selected concentration is highlighted in red. Places, where lanes not adjacent to each other on the gel had to be pasted together for comparison, have been marked with dotted lines.

 © 2023 The Authors

A

Different significance thresholds tested

Iter. no.	p<0.05	p<0.01	p<0.005	p<0.001
1	JAK → FGFR	JAK → FGFR	JAK → FGFR	JAK → FGFR
2	GSK3 → IGFR	GSK3 → IGFR	GSK3 → IGFR	GSK3 → IGFR
3	ACTR → ERK	ERK → MEK	ERK → RAF	BMP4R → FGFR
4	MEK→ RAF	BMP4R → FGFR	BMP4R → FGFR	ERK → RAF
5	JAK → GSK3	JAK → GSK3	BMP4R → SMAD2	JAK → GSK3
6	BMP4R → FGFR	BMP4R → SMAD2	JAK → GSK3	BMP4R → SMAD2
7	BMP4R → SMAD2	ACTR → RAS	ACTR → RAS	ACTR → BMP4R
8	GSK3 → LIFR	GSK3 → STAT3	LIFR → AKT	NA
9	JAK → BMP4R	LIFR → GSK3	GSK3 → LIFR	NA
10	LIFR → GSK3	JAK → BMP4R	JAK → BMP4R	NA

B

Categories of added links with different thresholds

Link	p<0.05	p<0.01	p<0.005	p<0.001
JAK → FGFR	**Yes**	**Yes**	**Yes**	**Yes**
GSK3 → IGFR	**Yes**	**Yes**	**Yes**	**Yes**
JAK → GSK3	**Yes**	**Yes**	**Yes**	**Yes**
BMP4R → FGFR	**Yes**	**Yes**	**Yes**	**Yes**
BMP4R → SMAD2	**Yes**	**Yes**	**Yes**	**Yes**
ERK → RAF	No	No	**Yes**	**Yes**
ERK→ MEK	No	**Yes**	No	No
MEK → RAF	**Yes**	No	No	No
ACTR → ERK	**Yes**	No	No	No
ACTR → RAS	No	**Yes**	**Yes**	No
ACTR → BMP4R	No	No	No	**Yes**
JAK → BMP4R	**Yes**	**Yes**	**Yes**	No
GSK3 → LIFR	**Yes**	No	**Yes**	No
GSK3 → STAT3	No	**Yes**	No	No
LIFR → GSK3	**Yes**	**Yes**	No	No
LIFR → AKT	No	No	**Yes**	No

C

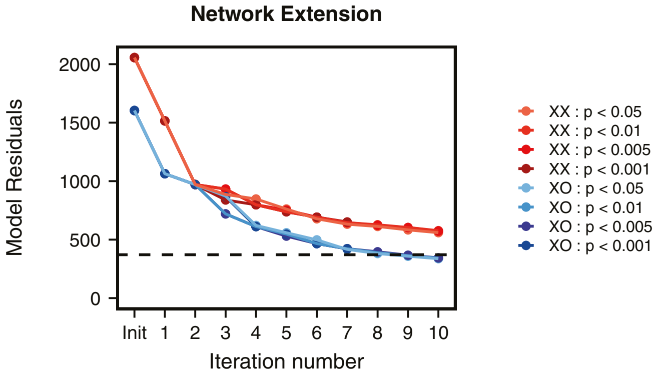

Figure EV2. Robustness of the network extension procedure.

A Links selected when model extension procedure is repeated with different *P*-value thresholds for the likelihood ratio test for the significance of added link.

B The links added when different *P*-value thresholds are used (listed in A) fall into 10 qualitatively distinct categories. The links that were deemed qualitatively similar have been given a common background color.

C Change in residuals of the XX and XO models when links are added as per the different *P*-value thresholds.

© 2023 The Authors

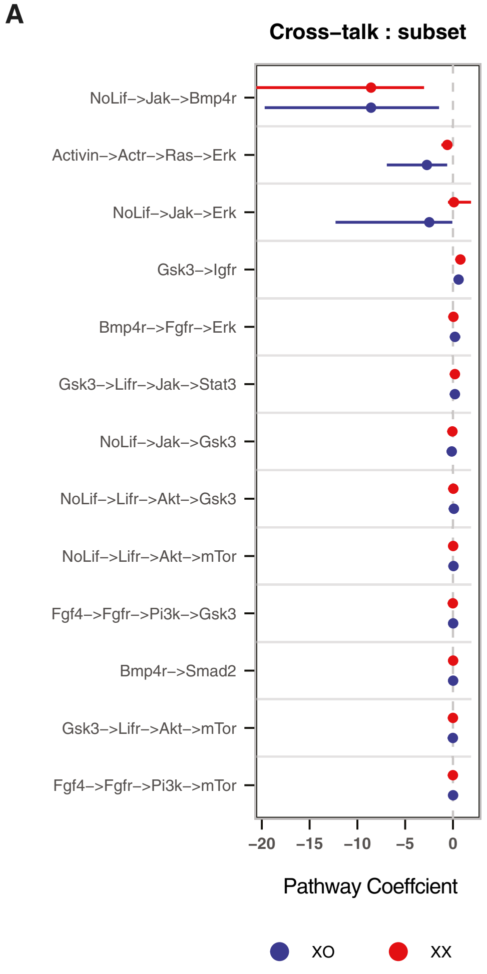

Figure EV3. Identifying links that have different strengths in the network for XX and XO cells.

A 95% confidence intervals of the composite parameters derived using profile likelihood for the paths capturing the important crosstalk in the completed networks. The cross-talk paths that span more than two linear canonical pathways are not shown.

 © 2023 The Authors

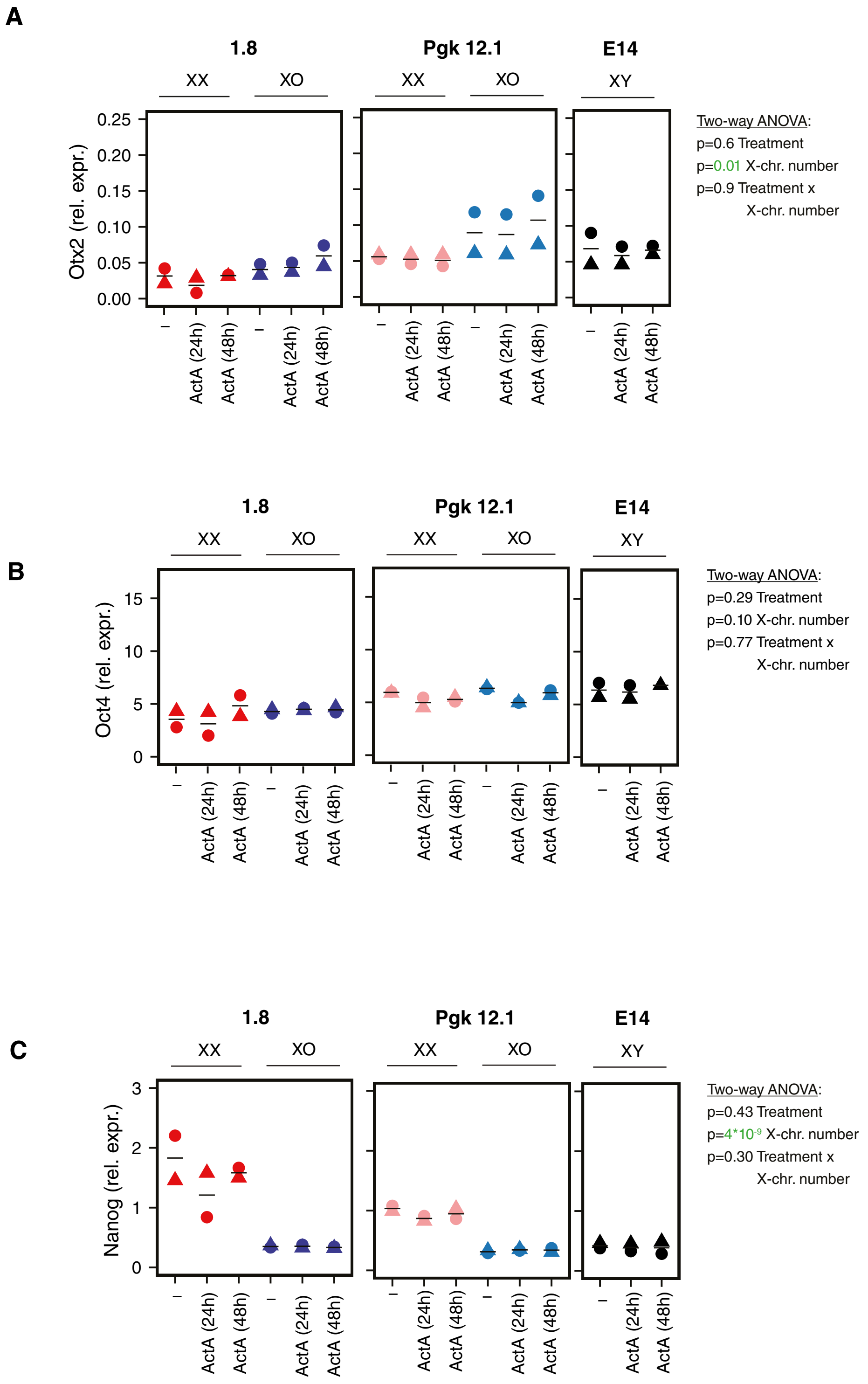

Figure EV4. Transcriptional response to Activin A treatment in XX, XO, and XY mESCs.

A–C Treatment of two XX/XO cell lines pairs (1.8, Pgk12.1) and XY mESCs (E14) with 30 ng/ml ActA for 24 or 48 h as indicated. Otx2 (A), Oct4 (B), and Nanog (C) expressions were assayed by qRT-PCR. The mean (lines) of two biological replicates (symbols) are shown. Results of a two-way ANOVA analyzing the effects of ActA treatment and X-chromosome number on the respective gene are reported, and $P < 0.05$ are colored in green.

Source data are available online for this figure.

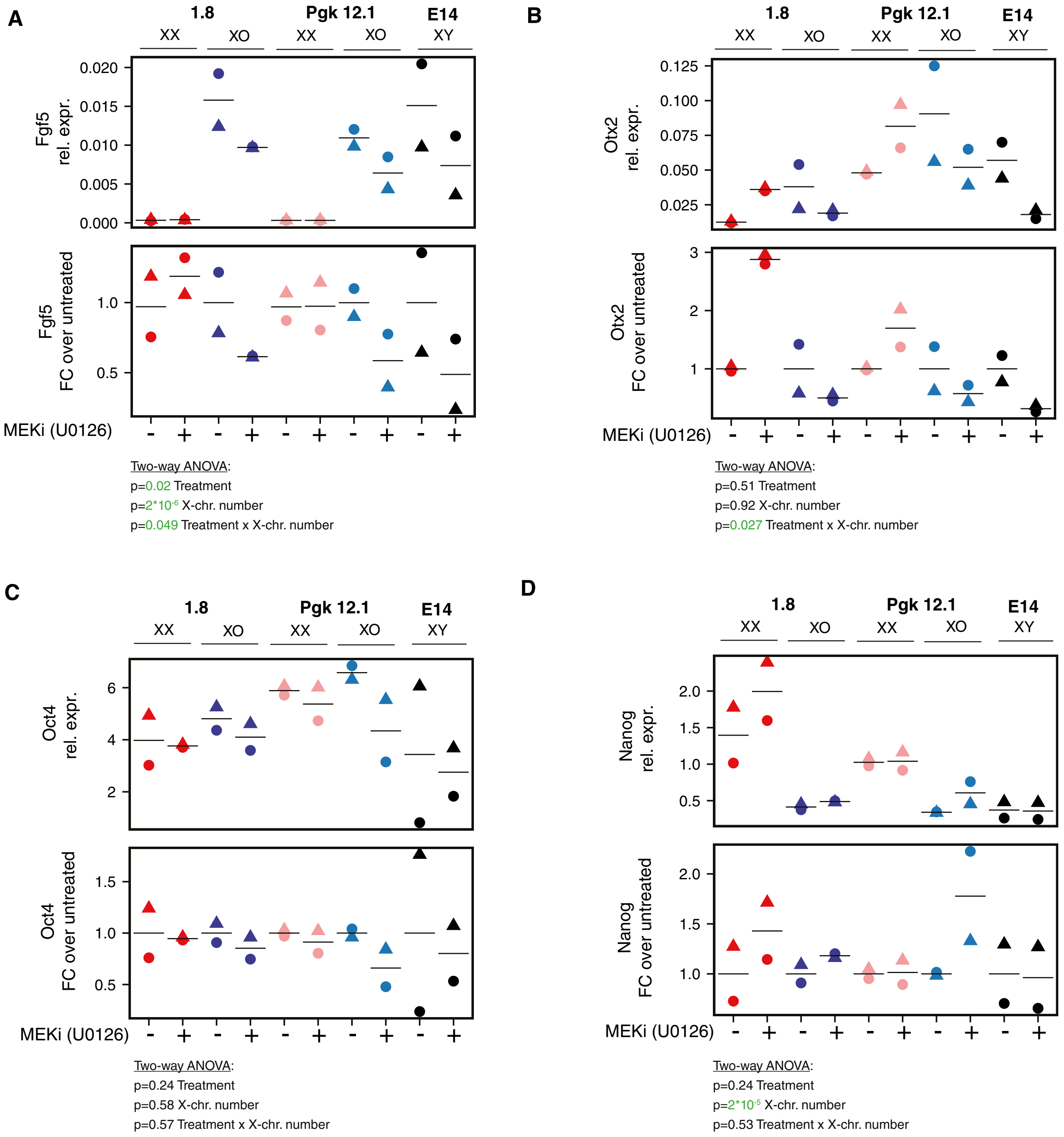

Figure EV5. Transcriptional response to MEKi treatment in XX, XO, and XY mESCs.

A–D Treatment of two XX/XO cell lines pairs (1.8, Pgk12.1) and XY mESCs (E14) 5 μM MEK inhibitor (U0126) for 24 h. Fgf5 (A), Otx2 (B), Oct4 (C), and Nanog (D) expressions were assayed by qRT-PCR. The mean (lines) of 2 biological replicates (symbols) are shown. Results of a two-way ANOVA analyzing the effects of MEKi treatment and X-chromosome number on the respective gene are reported, $P < 0.05$ are colored in green.

Source data are available online for this figure.

 © 2023 The Authors
