# Supplementary figures and images for: Modeling unveils sex differences of signaling networks in mouse embryonic stem cells
Source: Mol Syst Biol. 2023 Sep 21;19(11):e11510. doi: 10.15252/msb.202211510 (PMC10632733; doi:10.15252/msb.202211510)

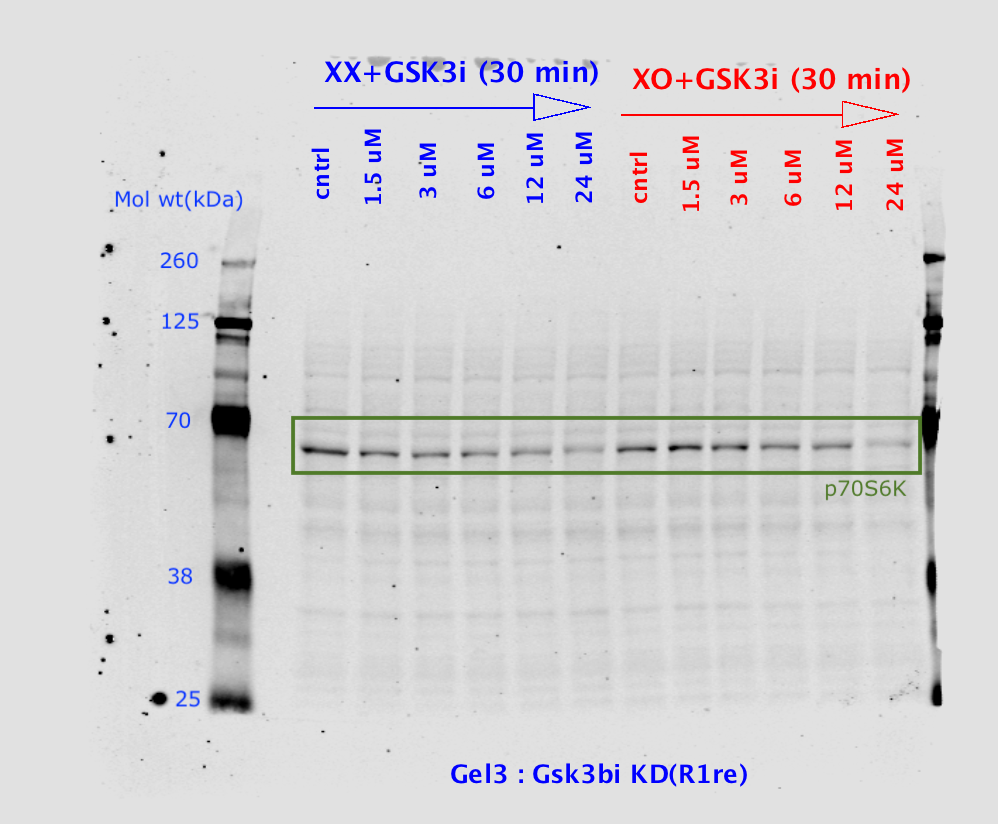

Supplement: Supplementary file 13 — Source Data for Figure 4 [file MSB-19-e11510-s013.zip › Figure4/4E/Fig4E_R1_Western_pP70S6K.tif]

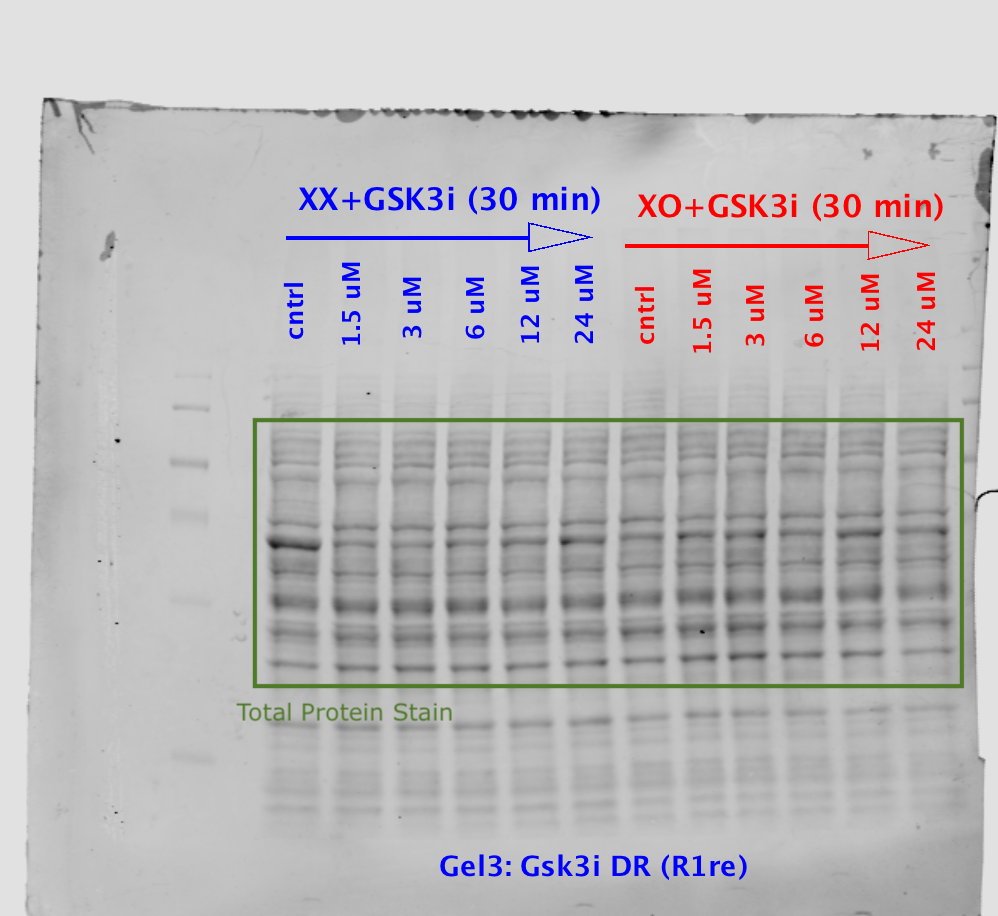

Supplement: Supplementary file 13 — Source Data for Figure 4 [file MSB-19-e11510-s013.zip › Figure4/4E/Fig4E_R1_Western_TotalProteinStain.tif]

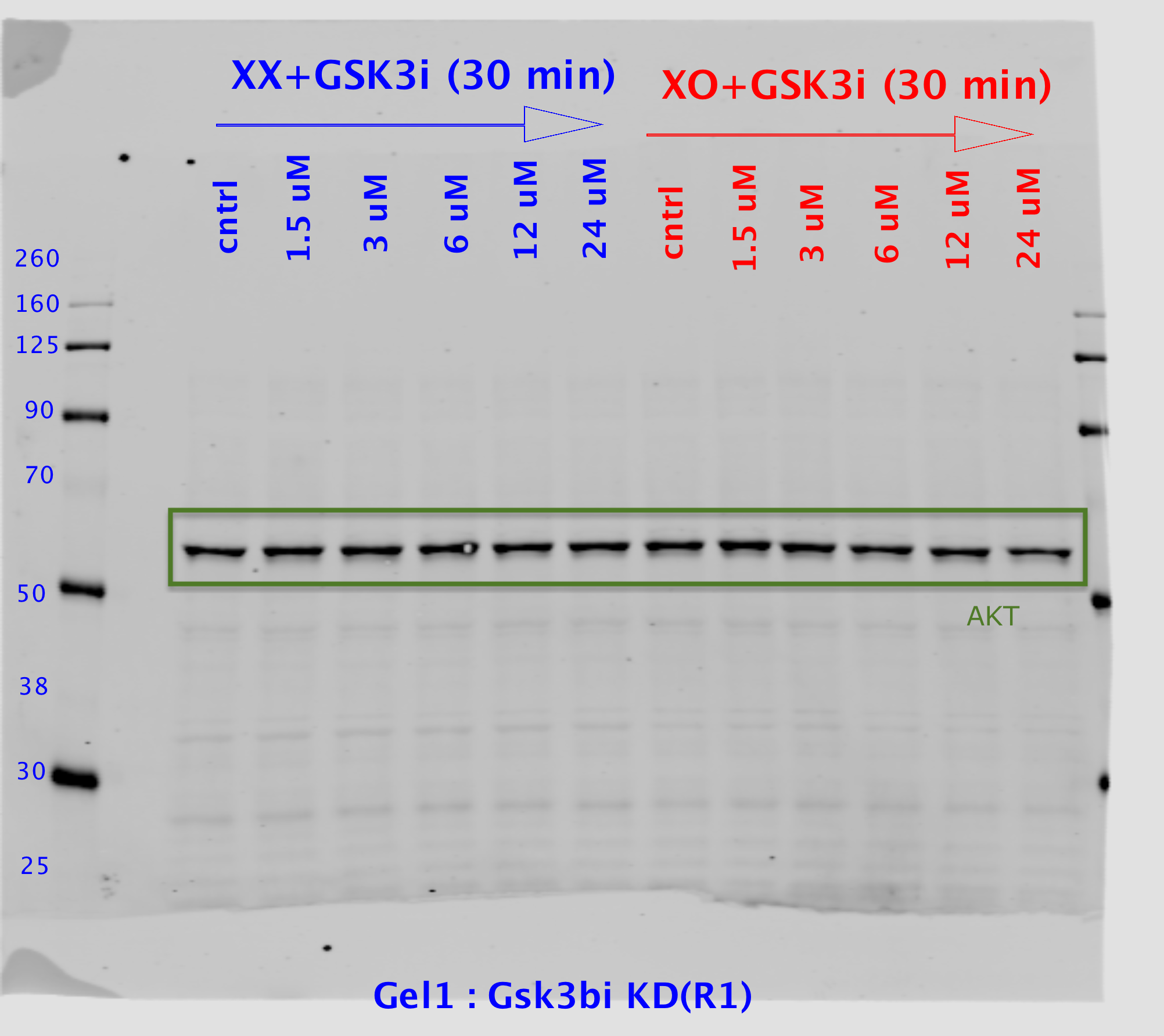

Supplement: Supplementary file 13 — Source Data for Figure 4 [file MSB-19-e11510-s013.zip › Figure4/4B/Fig4B_R1_Western_Akt.tif]

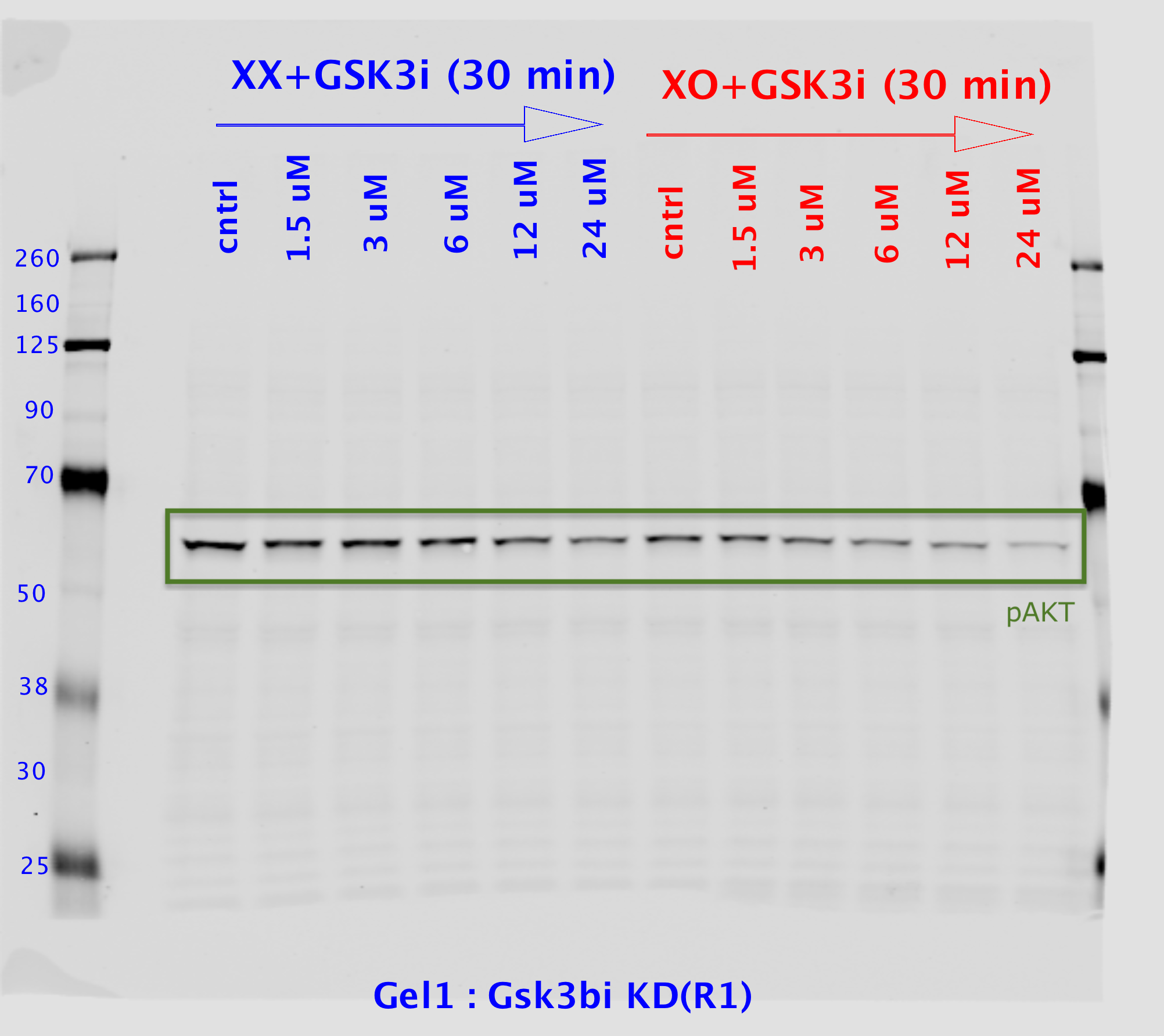

Supplement: Supplementary file 13 — Source Data for Figure 4 [file MSB-19-e11510-s013.zip › Figure4/4B/Fig4B_R1_Western_pAkt.tif]

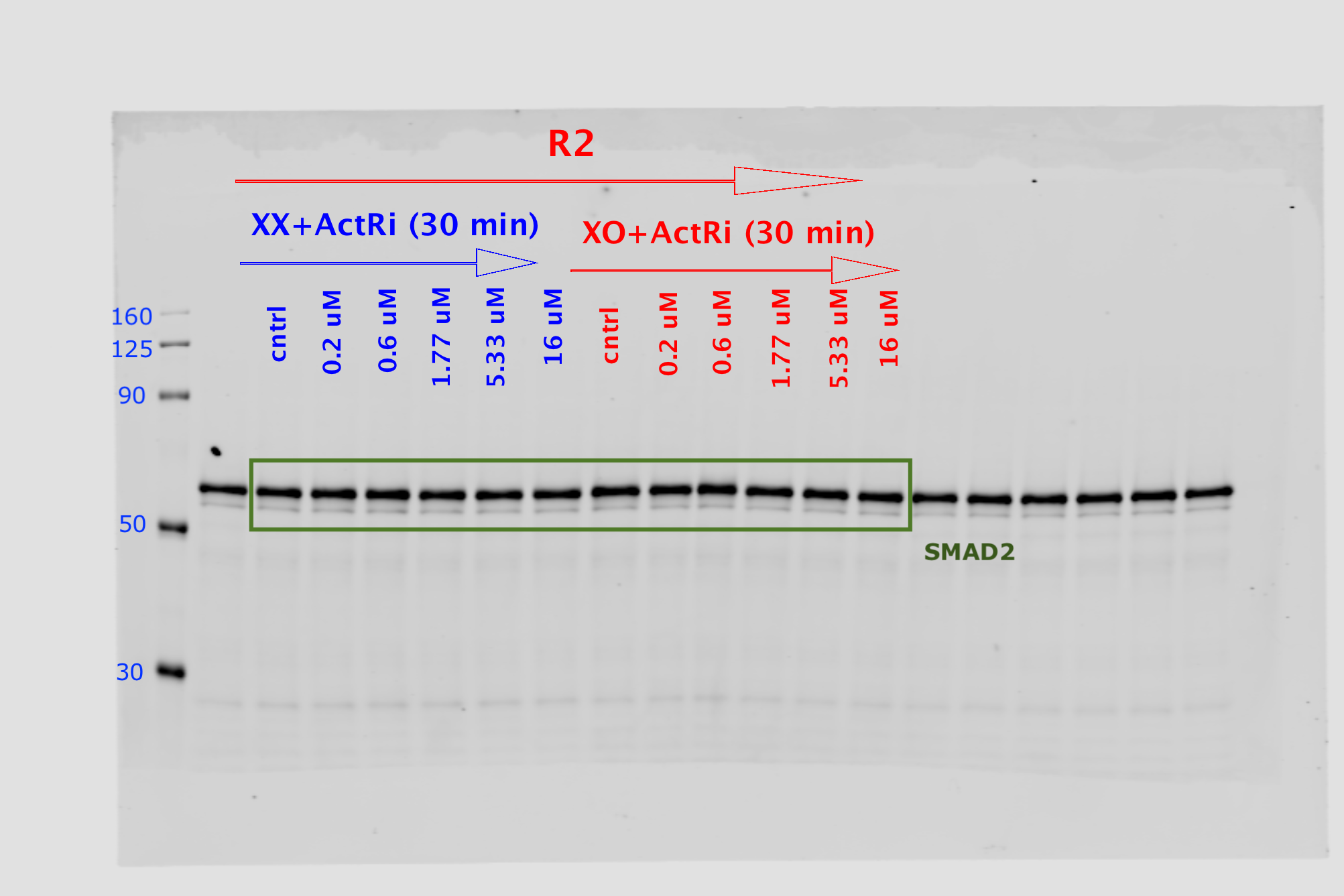

Supplement: Supplementary file 14 — Source Data for Figure 6 [file MSB-19-e11510-s014.zip › Figure6/6B/Fig6B_Lower_R2_ACTRi_Western_TotalSmad2.tif]

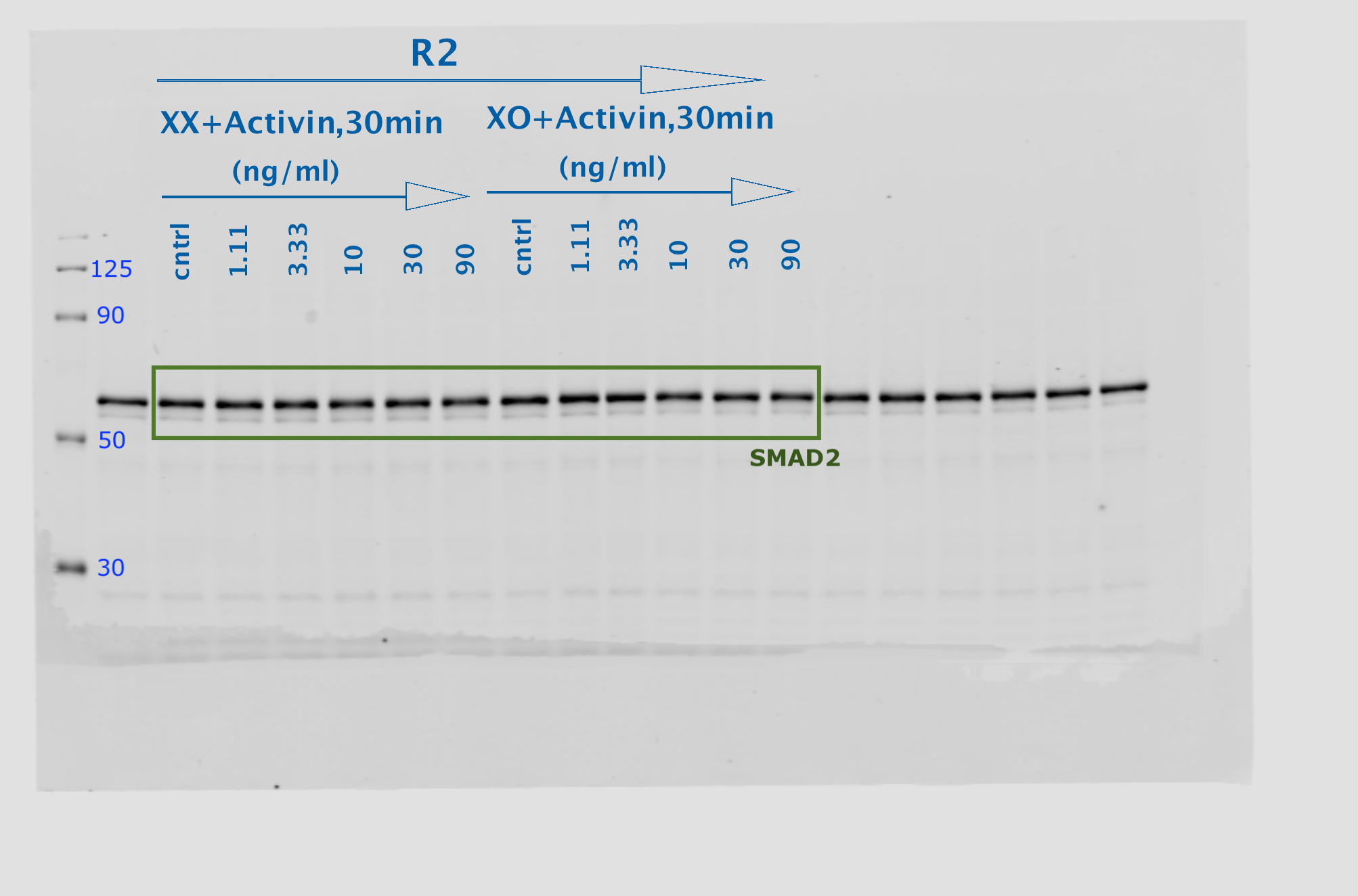

Supplement: Supplementary file 14 — Source Data for Figure 6 [file MSB-19-e11510-s014.zip › Figure6/6B/Fig6B_Upper_R2_ActA_Western_TotalSmad2.tif]

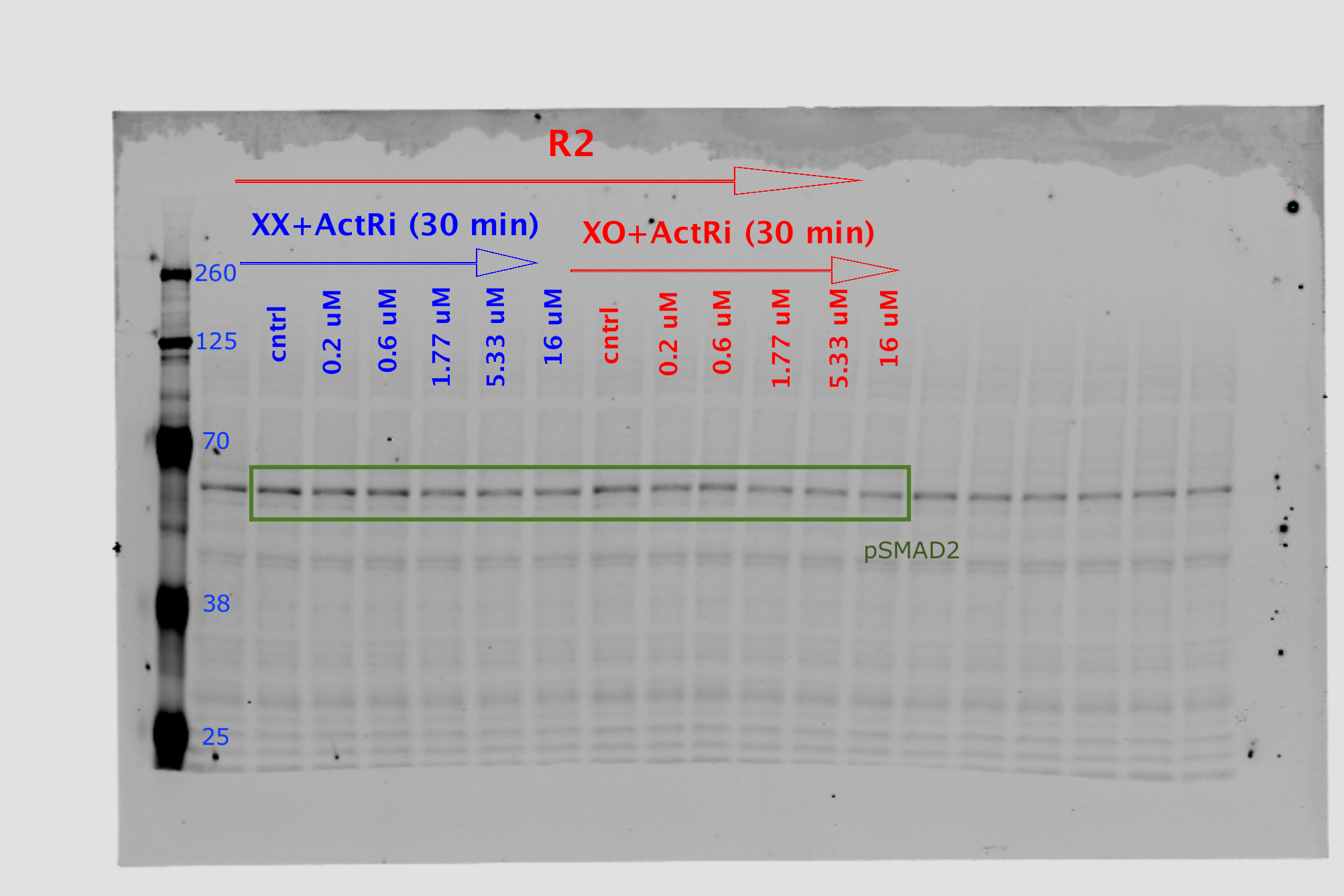

Supplement: Supplementary file 14 — Source Data for Figure 6 [file MSB-19-e11510-s014.zip › Figure6/6B/Fig6B_Lower_R2_ACTRi_Western_pSmad2.tif]

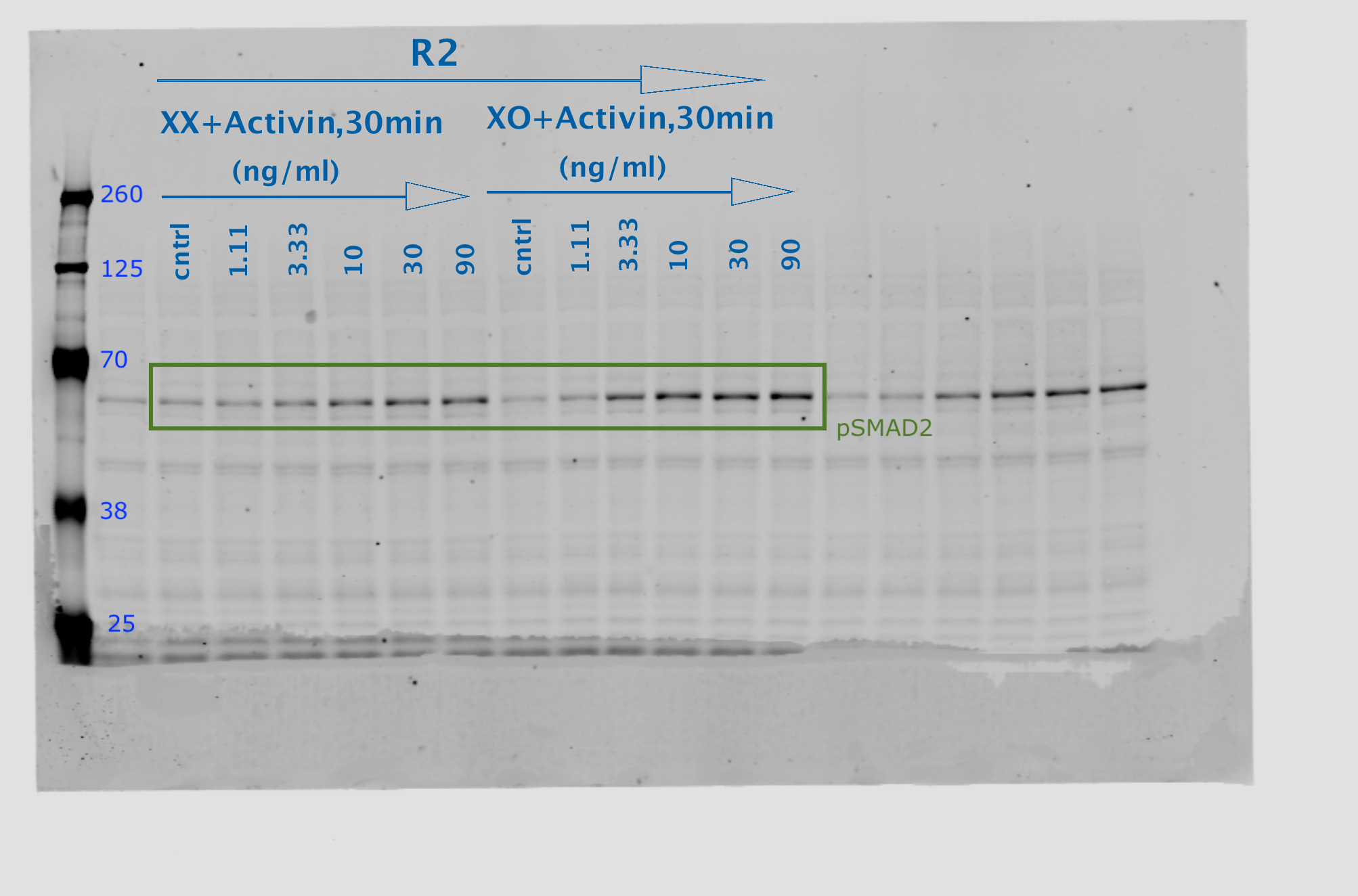

Supplement: Supplementary file 14 — Source Data for Figure 6 [file MSB-19-e11510-s014.zip › Figure6/6B/Fig6B_Upper_R2_ActA_Western_pSmad2.tif]

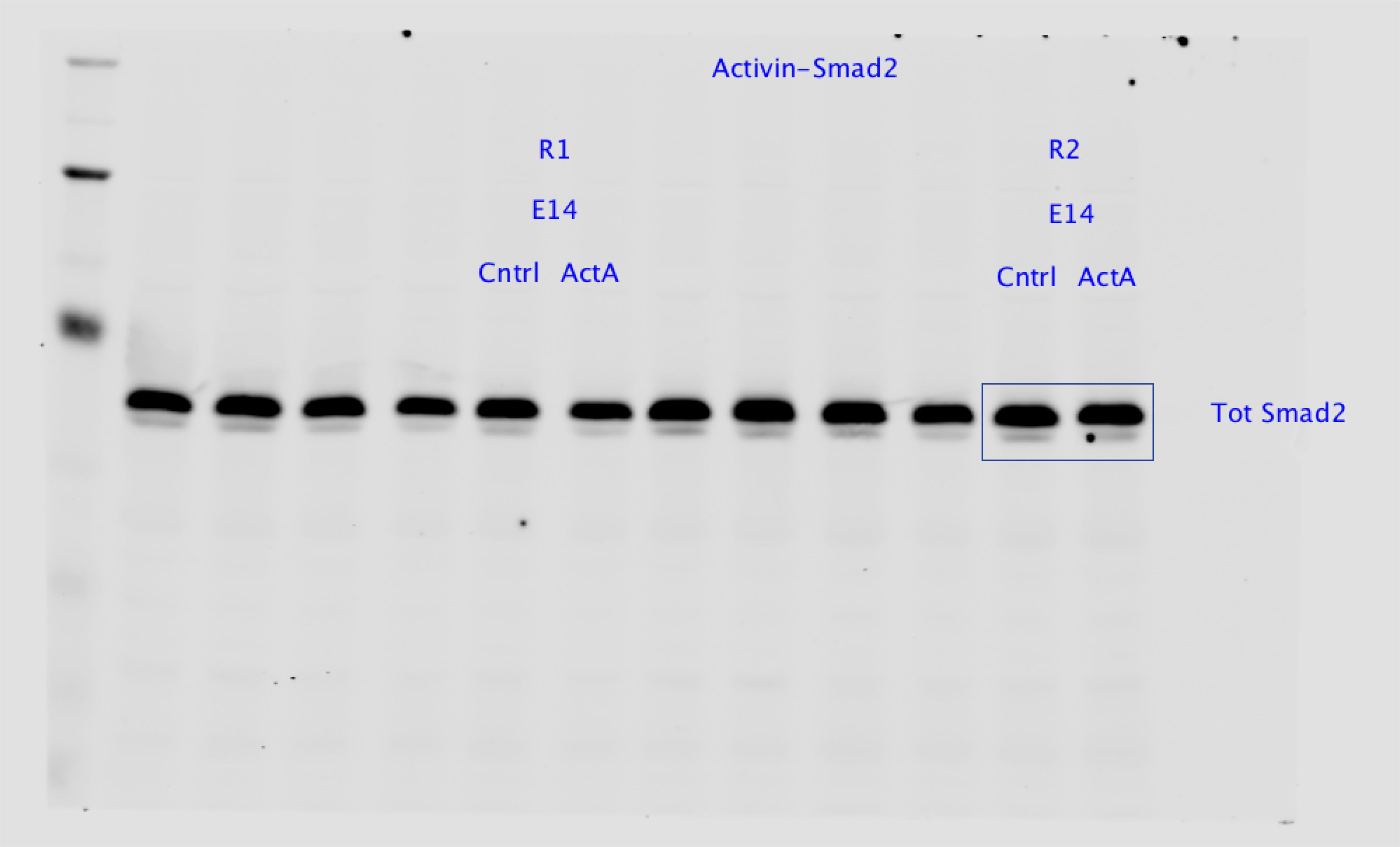

Supplement: Supplementary file 14 — Source Data for Figure 6 [file MSB-19-e11510-s014.zip › Figure6/6D/Fig6D_ActA_Tot_SMAD2_E14_R2.tif]

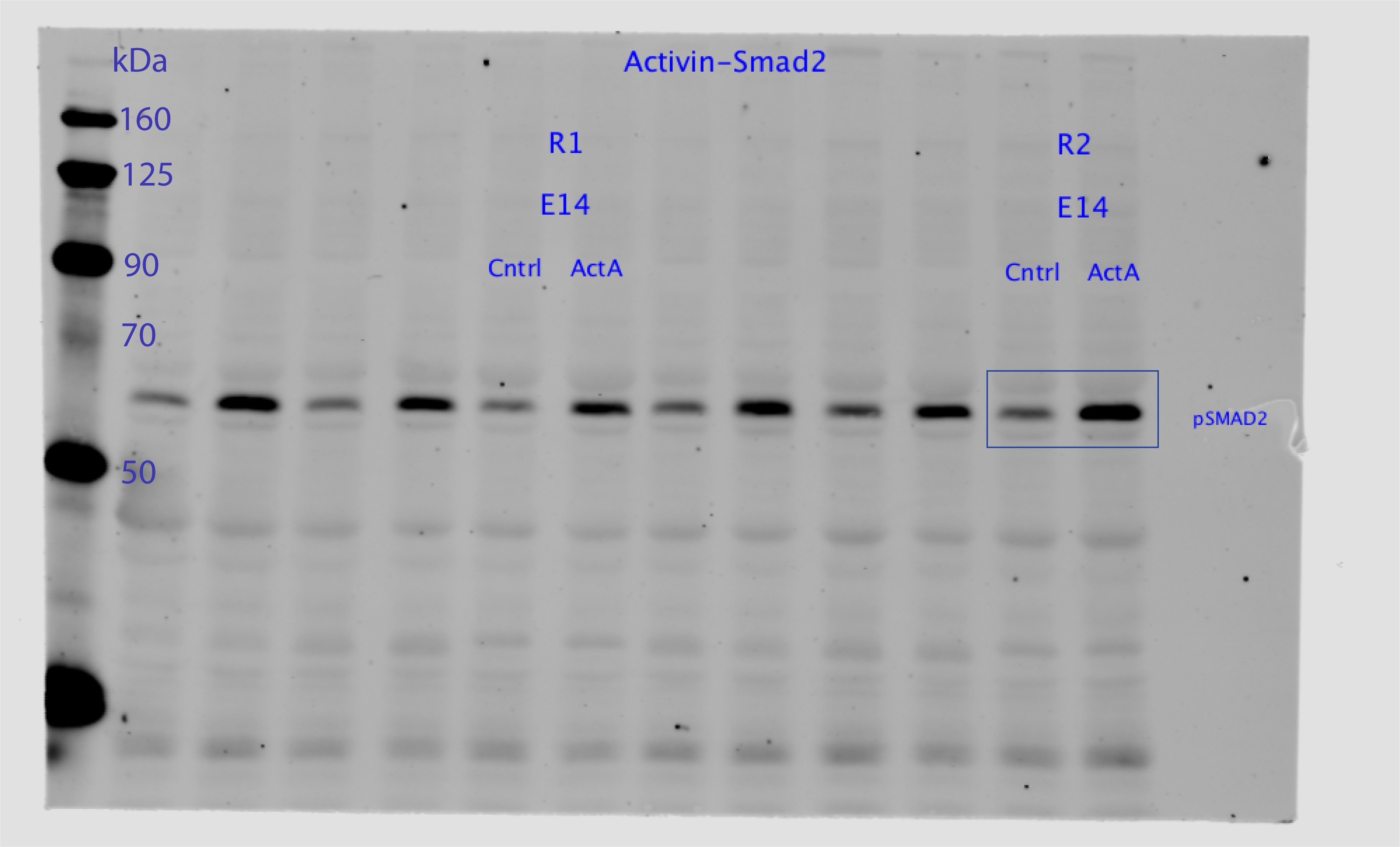

Supplement: Supplementary file 14 — Source Data for Figure 6 [file MSB-19-e11510-s014.zip › Figure6/6D/Fig6D_ActA_pSMAD2_E14_R2.tif]

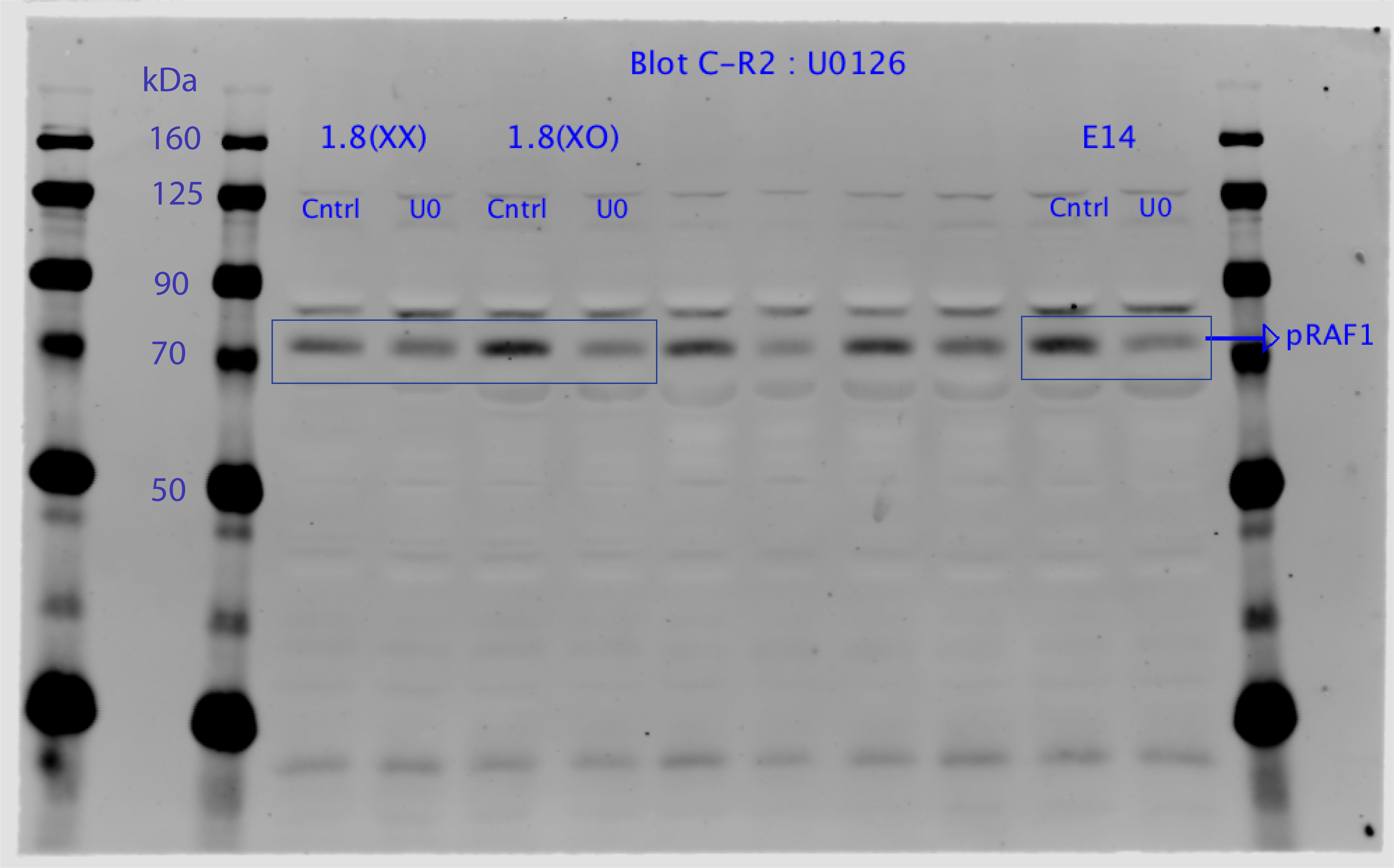

Supplement: Supplementary file 15 — Source Data for Figure 7 [file MSB-19-e11510-s008.zip › Figure7/7B/Fig7B_U0126_pRAF1_1.8_E14_R2.tif]

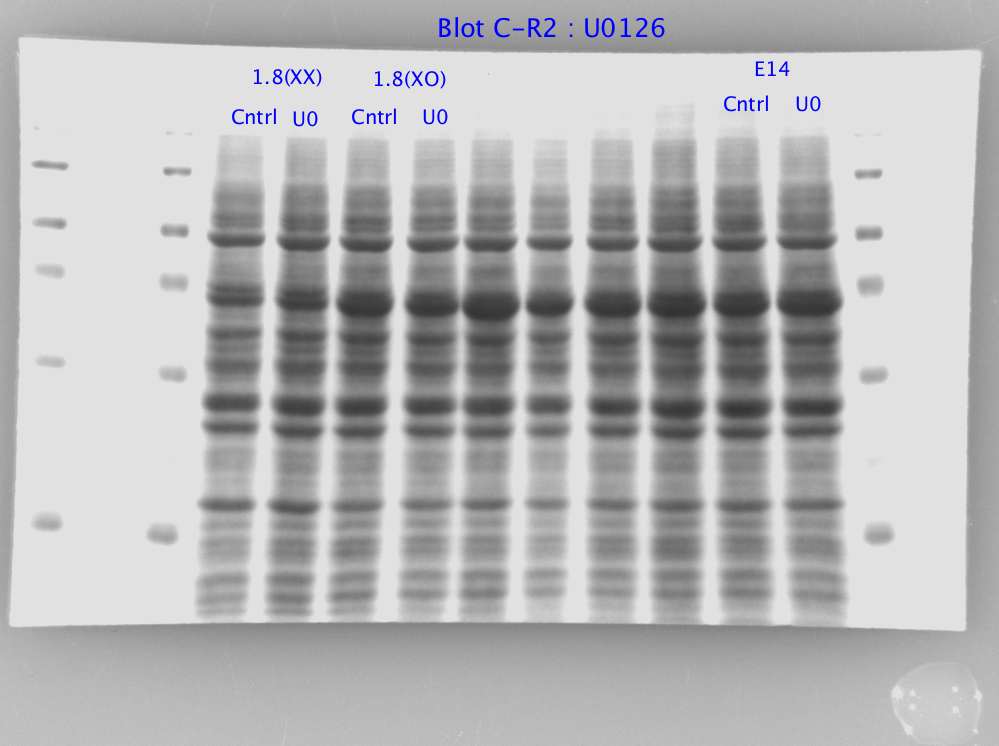

Supplement: Supplementary file 15 — Source Data for Figure 7 [file MSB-19-e11510-s008.zip › Figure7/7B/Fig7B_U0126_TPS_1.8_E14_R2.tif]

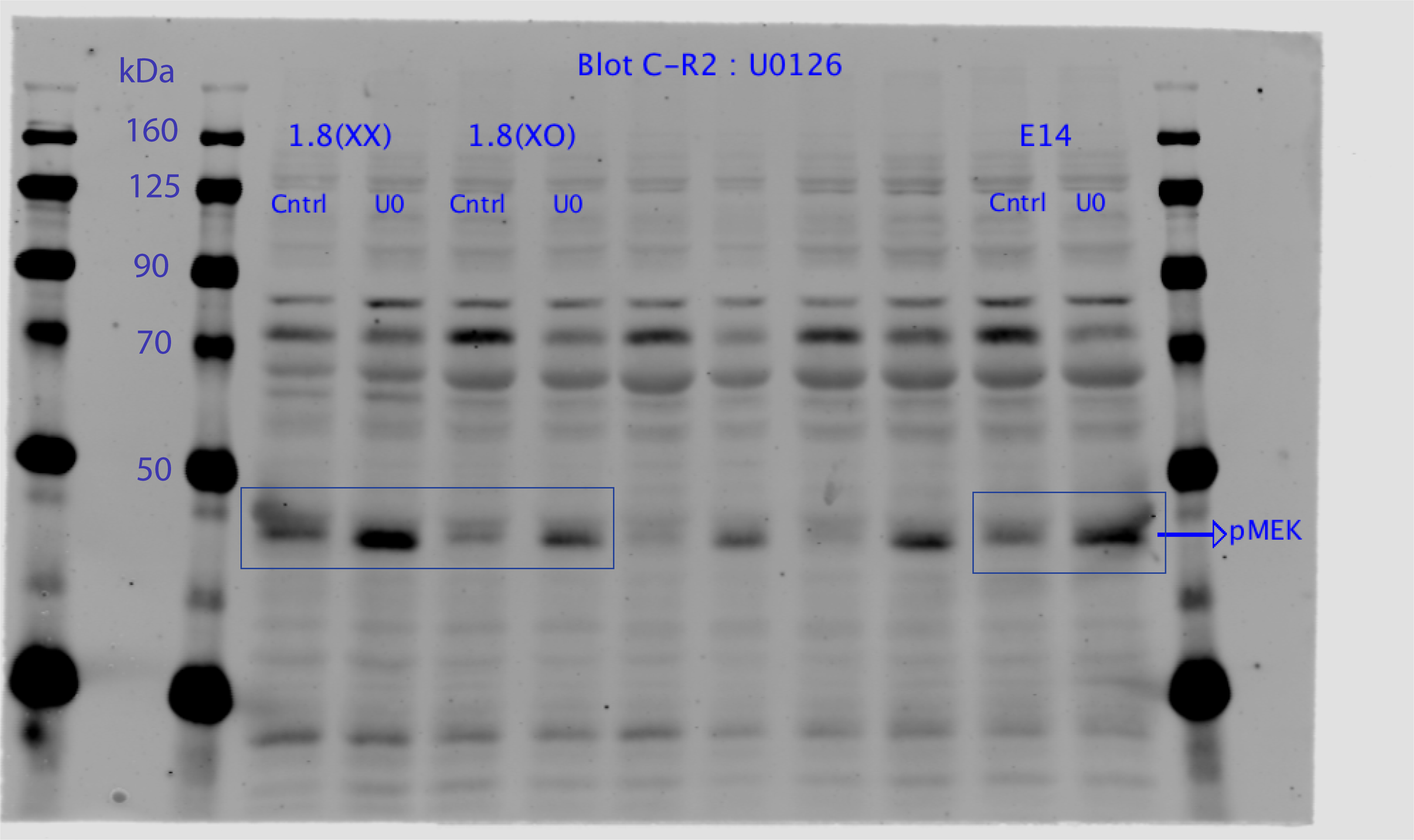

Supplement: Supplementary file 15 — Source Data for Figure 7 [file MSB-19-e11510-s008.zip › Figure7/7B/Fig7B_U0126_pMEK_1.8_E14_R2.tif]

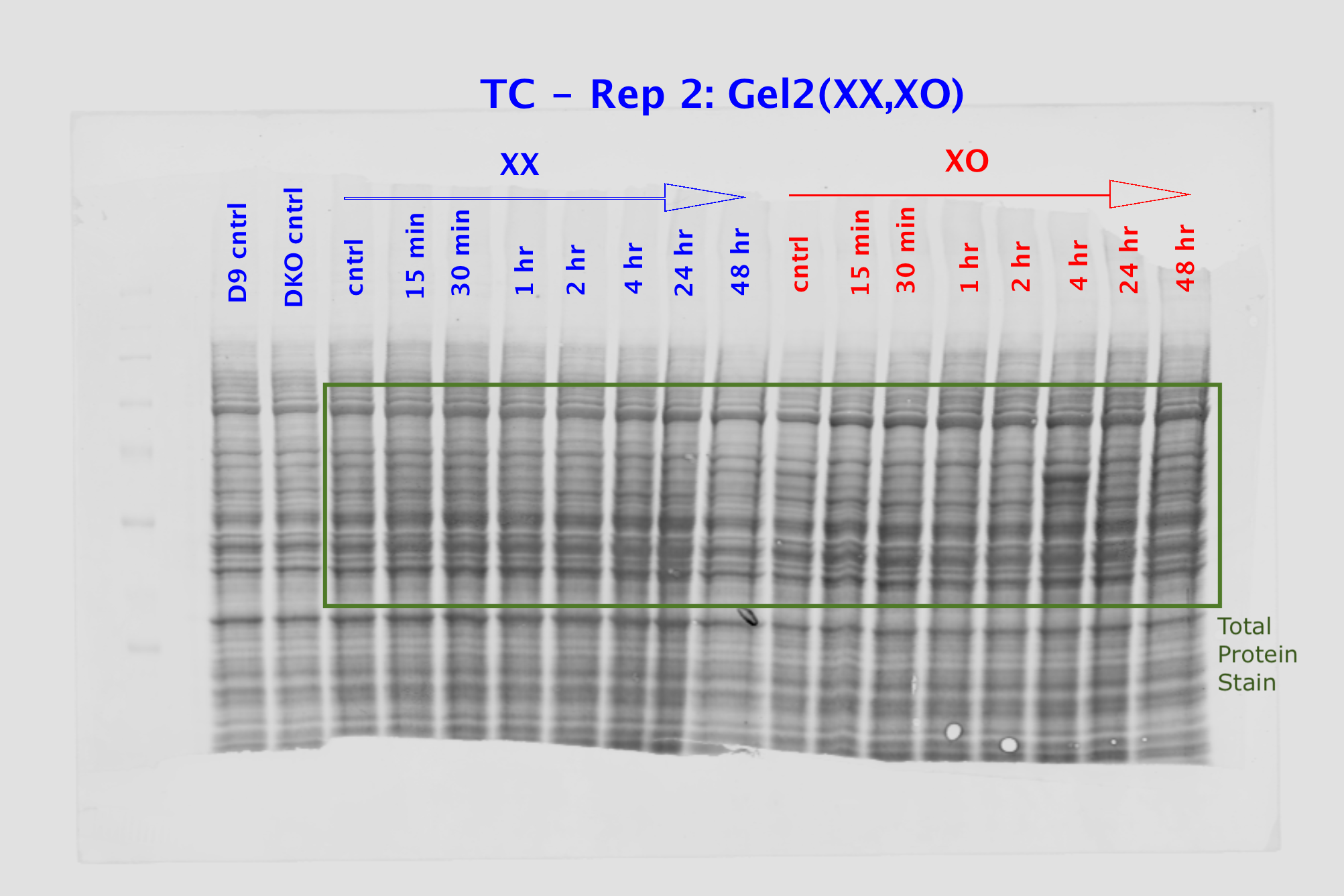

Supplement: Supplementary file 15 — Source Data for Figure 7 [file MSB-19-e11510-s008.zip › Figure7/7E/Fig7E_R2_TotalProteinStain_Western.tif]

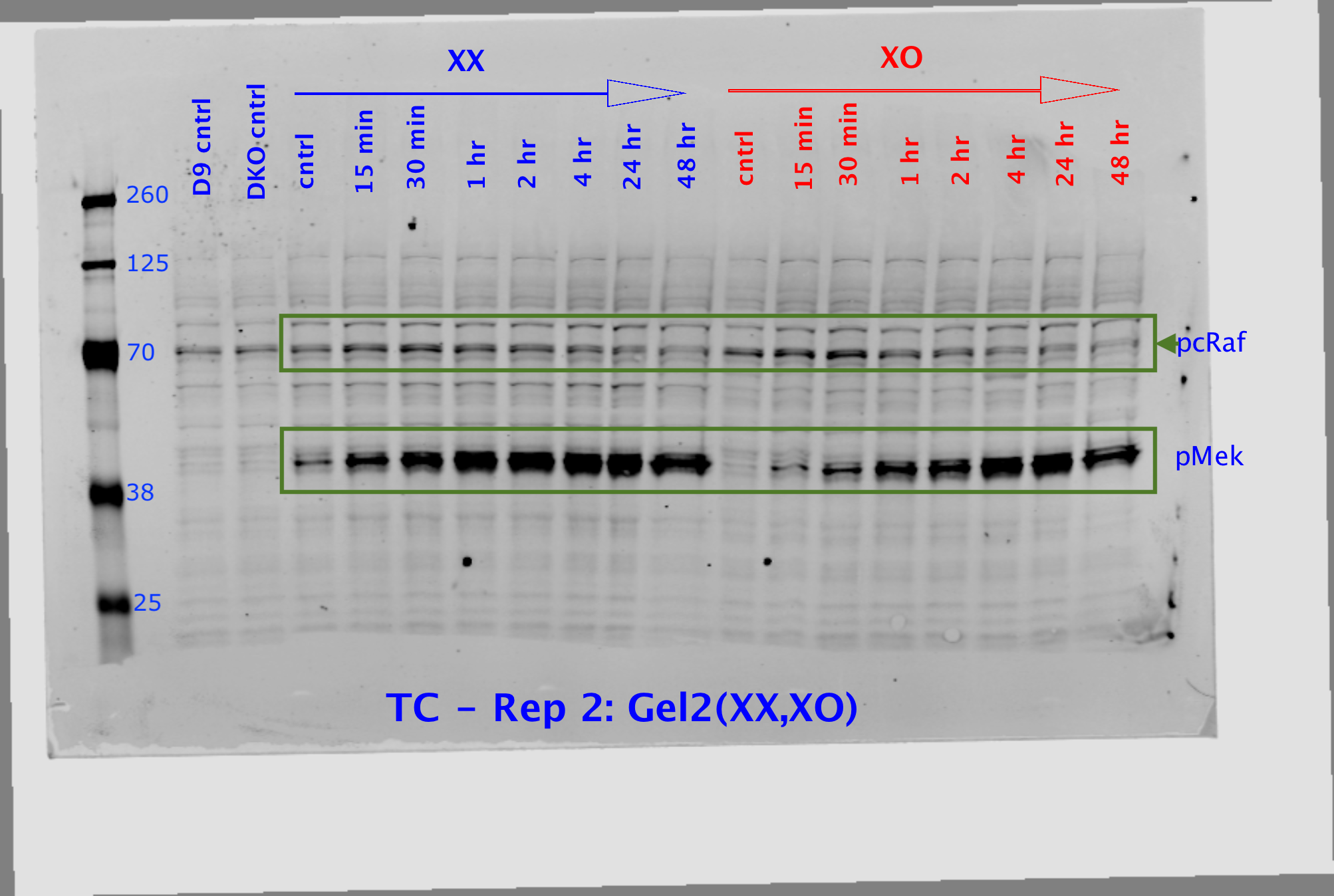

Supplement: Supplementary file 15 — Source Data for Figure 7 [file MSB-19-e11510-s008.zip › Figure7/7E/Fig7E_R2_pMek_pcRaf_Western.tif]

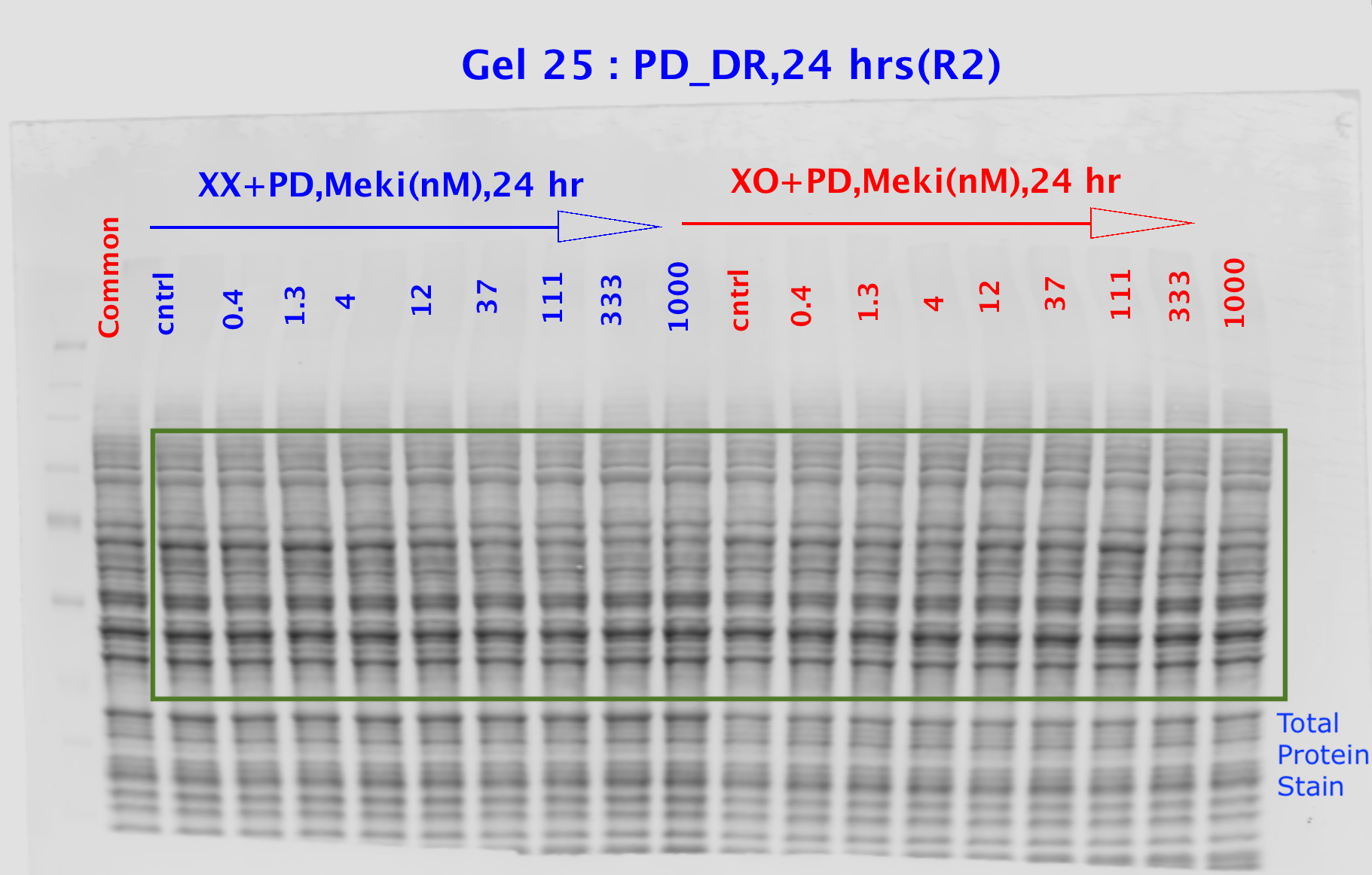

Supplement: Supplementary file 15 — Source Data for Figure 7 [file MSB-19-e11510-s008.zip › Figure7/7H/Fig7H_R2_TotalProteinStain_Western.png]

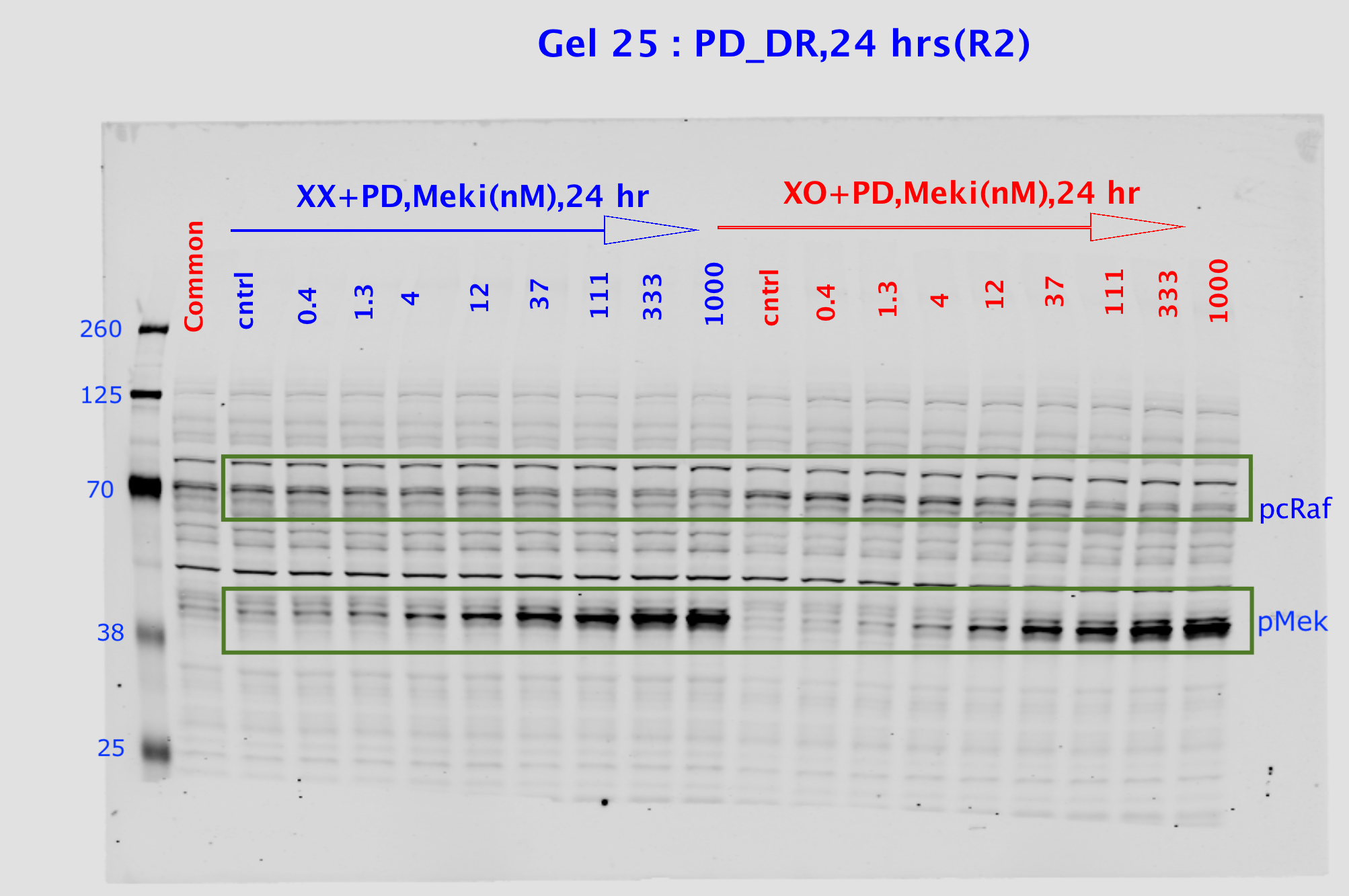

Supplement: Supplementary file 15 — Source Data for Figure 7 [file MSB-19-e11510-s008.zip › Figure7/7H/Fig7H_R2_pMek_pcRaf_Western.tif]
